# Using passenger mutations to estimate the timing of driver mutations and identify mutator alterations

**DOI:** 10.1186/1471-2105-14-363

**Published:** 2013-12-13

**Authors:** Ahrim Youn, Richard Simon

**Affiliations:** 1 Biometric Research Branch, National Cancer Institute, Bethesda, Maryland, USA

**Keywords:** Probabilistic modeling of tumor development, Estimating the order of mutations during tumorigenesis, Identifying mutator genes

## Abstract

**Background:**

Recent developments in high-throughput genomic technologies make it possible to have a comprehensive view of genomic alterations in tumors on a whole genome scale. Only a small number of somatic alterations detected in tumor genomes are driver alterations which drive tumorigenesis. Most of the somatic alterations are passengers that are neutral to tumor cell selection. Although most research efforts are focused on analyzing driver alterations, the passenger alterations also provide valuable information about the history of tumor development.

**Results:**

In this paper, we develop a method for estimating the age of the tumor lineage and the timing of the driver alterations based on the number of passenger alterations. This method also identifies mutator genes which increase genomic instability when they are altered and provides estimates of the increased rate of alterations caused by each mutator gene. We applied this method to copy number data and DNA sequencing data for ovarian and lung tumors. We identified well known mutators such as TP53, PRKDC, BRCA1/2 as well as new mutator candidates PPP2R2A and the chromosomal region 22q13.33. We found that most mutator genes alter early during tumorigenesis and were able to estimate the age of individual tumor lineage in cell generations.

**Conclusions:**

This is the first computational method to identify mutator genes and to take into account the increase of the alteration rate by mutator genes, providing more accurate estimates of the tumor age and the timing of driver alterations.

## Background

Recent developments in high-throughput genomic technologies are providing a comprehensive view of genomic alterations in tumors, including DNA copy number changes and nucleotide mutations on a whole genome scale. Although a large number of somatic alterations are detected in tumor genomes, only a small number of those are considered driver alterations which drive clonal expansion and invasion. Most of the somatic alterations appear to be passengers that are neutral for tumor cell selection [1]. Currently, most research efforts are put into distinguishing and analyzing driver alterations although an in-depth understanding of the driver alterations in the early stages of tumorigenesis has not emerged for most cancer types.

The passenger alterations can provide valuable information about the tumor. The number of passenger somatic alterations accumulated in the tumor can provide information about the approximate age of the tumor lineage, which is the number of cell divisions in the dominant clone’s lineage from the birth of the patient until the biopsy. Somatic alterations are acquired at each cell division with small probability, therefore, tumor samples which have undergone many cell divisions tend to accumulate many passenger alterations [2].

In addition to providing information on the age of the tumor lineage, passenger alterations can also give information about the approximate timing of the driver alterations occuring during tumorigenesis. Although the driver alterations and their order of occurrence differ among tumors, elucidating this information can be important for understanding tumorigenesis. Tumors evolve through a sequence of somatic driver alterations [3]. Mutations occur randomly and are selected for in cellular clonal evolution. For example, during early tumorigenesis, mutations which confer growth advantage may be selected for, however, as the tumor expands, mutations which give advantage in the condition of cellular crowding and substrate limitations due to reduced blood flow will be selected [4]. Early mutations may represent important therapeutic targets because they occur in all tumor clones and late mutations may play important roles in metastasis. Due to the importance of understanding the temporal order of driver alterations during tumorigenesis, several computational methods have been developed to estimate this order [5-9], but no previous methods have used passenger alterations for that purpose.

If a driver alteration occurs late during tumorigenesis, it will be found mainly in tumors with a large number of passenger somatic alterations. If it occurs early, the number of passenger alterations should be smaller. One important caveat, however, is that the rate of formation and accumulation of new passenger alterations may increase during tumorigenesis.

The most frequently observed genomic instability is chromosome instability (CIN), which refers to a high rate of chromosome structure alteration in cancer cells. Another form of genomic instability is characterized by increased frequencies of nucleotide mutations. Microsatellite instability (MIN), which is a special case of this genomic instability is characterized by the expansion or contraction of the number of oligonucleotide repeats present in microsatellite sequences [10].

A higher rate of nucleotide mutations or chromosome alterations during tumorigenesis is caused by alterations in genes that maintain genomic stability. These so called mutator genes which increase genomic instability when altered, are involved in the processes of DNA sysnthesis and repair, chromosome segregation, damage surveillance, cell cycle checkpoints, and apoptosis [11-13].

Since alterations of mutator genes increase the rate of alterations, the samples in which mutator genes are altered tend to accumulate many passenger alterations. Therefore, if one does not take into account the increase of the rate of alterations due to mutator genes, the timing of the mutator gene alterations as well as the tumor age will be overestimated.

Here, we propose a method which estimates the age of the tumor lineage and the timing of the driver alterations from the number of passenger alterations. The alterations include point mutations, short insertions and deletions detected in sequencing data and copy number alterations detected in copy number data. This method also identifies mutator genes that induce increase of chromosome alteration or point mutations and estimates the increased rate of chromosome alterations or point mutations caused by the mutator gene during tumorigenesis.

In the Methods section, we introduce the data types to which this method can be applied and then describe the probability model used. We then present the results obtained from applying the method to ovarian cancer data and lung cancer data in the Results and discussion section.

## Methods

### Data types

Our method can be applied to sequencing data as well as copy number data. Sequencing data provide point mutations and short insertions or deletions (INDEL). Copy number data provide copy number alterations (CNA), either deletions or amplifications spanning a range of chromosomal regions. To apply our method, we first need to distinguish driver from passenger alterations.

For sequencing data, we first define driver genes as those which are more frequently mutated than expected by the background mutation rate. There are various methods to find driver genes and we use the method of Youn *et al.*[14] in this paper. We define the mutations detected in driver genes as driver alterations and those detected in non-driver genes as passenger alterations.

For copy number data, we use the segmented copy number obtained from the circular binary segmentation algorithm [15]. The circular binary segmentation algorithm splits the chromosomes of each sample into contiguous regions of constant copy number taking into account the noise in the data. We only consider the segmented regions whose value of the *log*_2_ copy number change are larger than 1 or less than -1 as CNAs. Of these CNAs, we define driver and passenger CNAs as follows.

We define the CNAs which occur 10^6^ base pairs away from each end of any GISTIC region (the chromosomal regions that are focally amplified or deleted recurrently, found by the algorithm GISTIC [16]) as passenger CNAs. If there are multiple passenger CNAs of the same type (amplification or deletion) that are close to each other (less than 10^5^ base pairs), we merge them.

We define the CNAs which overlap the GISTIC region of the same type (amplification or deletion) for longer than two thirds of the region as driver CNAs. In other words, we say that a sample contains an amplified driver CNA associated with a given focally amplified GISTIC region if the amplified segment (segment whose *log*_2_ copy number change is larger than 1) in the sample overlaps more than two thirds of the amplified GISTIC region. Similarly, a sample contains a deleted driver CNA associated with a given focally deleted GISTIC region if the deleted segment (segment whose *log*_2_ copy number change is less than −1) in the sample overlaps more than two thirds of the deleted GISTIC region.

### Probability model

For each tumor sample *i*, we know the number of passenger somatic alterations *N*_*i*_, the age of the patient *S*_*i*_, whether an alteration occurred in driver gene/region *j* or not (denoted by *A*_*i*,*j*_=1or 0) and whether it is germline or somatic (denoted by *G*_*i*,*j*_=1or 0).

From these data, we want to infer when the driver gene/region *j* alters in sample *i* and if altered, how much it increases the alteration rate of other genes or regions. We also want to estimate the age of tumor lineage *T*_*i*_. We define it as the number of cell divisions between the birth of the patient and the biopsy of the tumor in the lineage containing the founder cell of the dominant clone for sample *i*. We will use the Bayesian probabilitstic model defined below.

**Figure 1:**
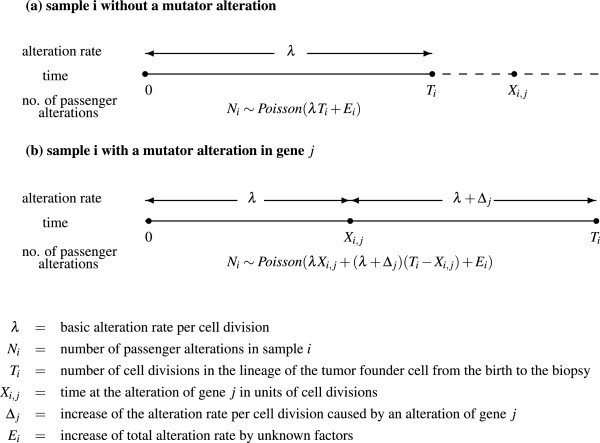
**Probability model for generation of passenger somatic alterations.(a)** for samples without mutator alterations, **(b)** for samples with a mutator alteration.

We model the accumulation of passenger somatic alterations in the lineage of tumor founder cell by a Poisson process. In the tumor cell lineage, we assume that new passenger alterations are acquired with rate *λ* at each cell division. Therefore, for the cell which has gone through *T*_*i*_ cell divisions, *N*_*i*_ follows Poisson distribution with rate *λT*_*i*_ if the alteration rate stays constant. (Figure 1(a)) In order to permit the increase of alterations by unknown factors such as exposure to mutagens by smoking or UV radiation, we add *E*_*i*_ and therefore, the number of passenger somatic alterations *N*_*i*_ follows a Poisson distribution with rate *λT*_*i*_+*E*_*i*_

When a driver gene or region *j* is altered in sample *i* (*A*_*ij*_=1), we assume that it increases the alteration rate by *Δ*_*j*_. It is positive if the gene/region is a mutator and 0 otherwise (Figure 1(b)). Suppose the alteration of the driver gene or region *j* occurred in sample *i* at time *X*_*i*,*j*_. We assume the increase *Δ*_*j*_ is independent of when the driver *j* is altered. Then until the time *X*_*i*,*j*_, the alteration rate per cell division is *λ* and after that, it becomes *λ*+*Δ*_*j*_. Therefore the number of passenger alterations follows a Poisson distribution with rate *λX*_*i*,*j*_+(*λ*+*Δ*_*j*_)(*T*_*i*_−*X*_*i*,*j*_)+*E*_*i*_=*λT*_*i*_+*Δ*_*j*_(*T*_*i*_−*X*_*i*,*j*_)+*E*_*i*_.

In general, when there are multiple driver genes/regions *j*∈*J*, each of which increases the alteration rate by *Δ*_*j*_, the number of passenger somatic alterations follows a Poisson distribution with rate $\lambda T_{i}+\underset{j\in J}{\sum }\Delta_{j}(T_{i}-X_{i,j})+E_{i}$ (The derivation of this is provided in the Additional file 1). This means that the alteration of each driver gene/region *j* increases the average number of passenger alterations accumulated in the sample by *Δ*_*j*_(*T*_*i*_−*X*_*i*,*j*_) additively. The value of *X*_*i*,*j*_ is unknown, but the values of *A*_*i*,*j*_ and *G*_*i*,*j*_ give some information about *X*_*i*,*j*_ since *A*_*i*,*j*_=1 implies *X*_*i*,*j*_≦*T*_*i*_ and *A*_*i*,*j*_=0 implies *X*_*i*,*j*_>*T*_*i*_. Also, *G*_*i*,*j*_=1 implies *X*_*i*,*j*_=0 since the alteration existed from the birth of the patient. *G*_*i*,*j*_=0 implies *X*_*i*,*j*_>0.

Since we cannot estimate *X*_*i*,*j*_ and *T*_*i*_ for each sample separately, we use a Bayesian approach and assume a prior distribution for *X*_*i*,*j*_ and *T*_*i*_. We assume *T*_*i*_ follows a Gamma distribution with an unknown shape and rate parameter *α*,*β*. We restrict the range of values it can assume for each sample to be between 50 and the age of the patient *S*_*i*_ divided by the tumor cell division time *r* for the specific tissue. This is because the number of cell divisions in the tumor lineage is unlikely to be less than 50 or larger than *S*_*i*_/*r* since cell divides most frequently after the onset of neoplasia in the lineage of the founder cell.

We assume *X*_*i*,*j*_ follows a Gamma distribution, however since it is possible that the alteration may never occur, we assume Pr(*X*_*i*,*j*_=*∞*)=*p*_*j*_>0. When 0<*X*_*i*,*j*_<*∞*, we assume *X*_*i*,*j*_ follows a Gamma distribution with shape and rate parameter *α*_*j*_,*β*_*j*_. We assume *E*_*i*_ follows an exponential distribution with a parameter *ρ*.

The rate of alteration *λ* differs for nucleotide mutations (point mutations and short INDELs) detected in sequencing data and CNAs detected in copy number data. The rate of nucleotide mutations per cell division *λ*^*M* *U* *T*^ is calculated using the experimentally obtained mutation rate per cell division and per base pair, 10^−9^[17,18].

$$\;\begin{array}{l} \lambda^{\text{MUT}}=\;10^{-9}\\ \;\;\times \text{number of base pairs sequenced for non-driver genes} \end{array}$$

The rate of CNAs per cell division, *λ*^*C* *N* *A*^ is unknown. The ratio of *λ*^*M* *U* *T*^ vs. *λ*^*C* *N* *A*^ is,

$$\;\begin{array}{l} R=\frac{\lambda^{\text{CNA}}}{\lambda^{\text{MUT}}}=\frac{\text{rate of CNAs per cell division}}{\text{rate of mutations per cell division}}\\ \;=\frac{\text{no. of CNAs}/\text{no. of cell divisions}}{\text{no. of mutations}/\text{no. of cell divisions}}=\frac{\text{no. of CNAs}}{\text{no. of mutations}}\\ \;≅\frac{\text{average no. of passenger CNAs per sample}}{\text{average no. of passenger mutations per sample}} \end{array}$$

Therefore, we estimate *λ*^*C* *N* *A*^ as *R*·*λ*^*M* *U* *T*^.

The unknown values of the parameters *α*,*β*,*α*_*j*_,*β*_*j*_,*Δ*_*j*_,*p*_*j*_,*ρ* are estimated by maximizing the likelihood of the observed data: the number of passenger somatic alterations *N*_*i*_ and occurrences of driver alterations *j* in sample *i* (*A*_*i*,*j*_) given their germline status (*G*_*i*,*j*_) and the age of the patient *S*_*i*_.

For given values of the times *x*_*i*,*j*_ of alterations of gene/region *j*∈*J* and the age of the tumor lineage *t*_*i*_, the number of passenger somatic alterations *N*_*i*_ in sample *i* would have a Poisson distribution with mean

$$\mu \;\left(x_{i,k},,,t_{i},,,e_{i}\right)=\underset{k,A_{i,k}=1}{\sum }\Delta_{k}\left(t_{i},-,x_{i,k}\right)+\lambda t_{i}+e_{i}$$

Then, the likelihood of observing *N*_*i*_ and *A*_*i*,*j*_ given their germline status *G*_*i*,*j*_ and age of the patient *S*_*i*_ is obtained by integrating Poisson (*n*_*i*_;*μ*(*x*_*i*,*k*_,*t*_*i*_,*e*_*i*_)) times probability density functions of *X*_*i*,*j*_, *T*_*i*_, *E*_*i*_ over the ranges of *X*_*i*,*j*_, *T*_*i*_ and *E*_*i*_ corresponding to *A*_*i*,*j*_=*a*_*i*,*j*_,*G*_*i*,*j*_=*g*_*i*,*j*_,∀*j*. When *G*_*i*,*j*_=1, *X*_*i*,*j*_ is zero. When *G*_*i*,*j*_=0, *X*_*i*,*j*_ is between 0 and *T*_*i*_ if *A*_*i*,*j*_=1; otherwise *X*_*i*,*j*_ is larger than *T*_*i*_. *T*_*i*_ takes values from 50 to the age of the patient *i* divided by the tumor cell division time r and *E*_*i*_ takes values from 0 to infinity. For the derivation of the likelihood function and the details of parameter estimation, see the Additional file 1.

This model can be applied to both sequencing and copy number data. For sequencing data, the number of passenger somatic mutations for sample *i*, $N_{i}^{\text{MUT}}$ is assumed to follow a Poisson distribution with rate $\lambda^{\text{MUT}}T_{i}+\underset{j\in J}{\sum }\Delta_{j}^{\text{MUT}}(T_{i}-X_{i,j})+E_{i}^{\text{MUT}}$ where *λ*^*M* *U* *T*^ is the basic nucleotide mutation rate and $\Delta_{j}^{\text{MUT}}$ is the increase of nucleotide mutation rate by the alteration of driver *j*. For copy number data, the number of passenger somatic CNAs, $N_{i}^{\text{CNA}}$ is assumed to follow a Poisson distribution with rate $\lambda^{\text{CNA}}T_{i}+\underset{j\in J}{\sum }\Delta_{j}^{\text{CNA}}(T_{i}-X_{i,j})+E_{i}^{\text{CNA}}$ where *λ*^*C* *N* *A*^ is the basic CNA rate and $\Delta_{j}^{\text{CNA}}$ is the increase of CNA rate by the alteration of driver *j*.

With these parameters, we can obtain the posterior mean of *T*_*i*_ and *X*_*i*,*j*_ for each sample *i* given the data *N*_*i*_,*A*_*i*,*j*_ and *G*_*i*,*j*_. Also, using the posterior mean of *X*_*i*,*j*_, we can order the sequence of driver alterations which occurred for each sample *i*. In the Results and discussion section, we present the result obtained for ovarian and lung cancer data.

## Results and discussion

### Ovarian cancer data

We applied our method to the ovarian cancer data from The Cancer Genome Atlas (TCGA) [19], which analysed DNA copy number and whole exome sequences in 316 high-grade serous ovarian adenocarcinomas.

We first identified driver genes by applying the method of Youn *et al.*[14] to the whole exome sequencing data. We further select genes mutated in more than ten samples and obtained CSMD3, FAT3, NF1, TP53, USH2A, BRCA1 and BRCA2. The genes BRCA1 and BRCA2 have somatic mutations in 11 and 10 samples, but they have germline mutations in 27 and 20 samples, respectively.

**Figure 2:**
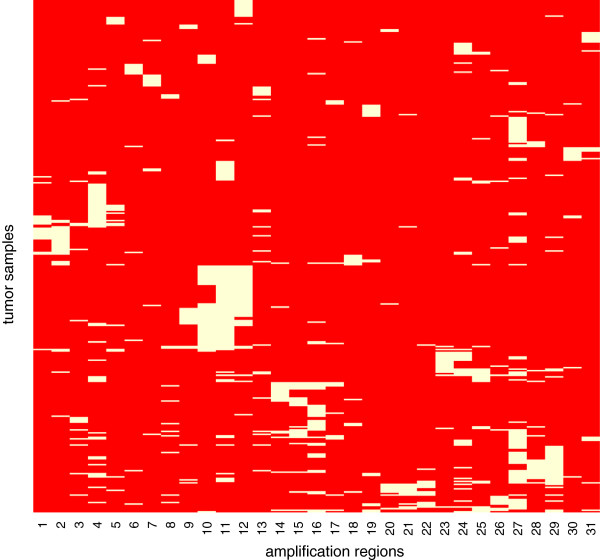
**Heatmap of amplification patterns of the significantly amplified regions in ovarian cancer found by GISTIC.** Columns represent amplified regions and rows represent tumor samples. The yellow color indicates that the region is amplified in the corresponding tumor samples. Columns are sorted by their chromosome locations.

Second, we used GISTIC to identify 63 regions of focal amplification and 50 regions of focal deletion from the copy number data. Although GISTIC identified 113 driver regions, we found that many of the regions show correlated pattern of alterations as shown in Figure 2. It is a heatmap of amplification patterns of focal amplification regions amplified in more than ten samples. Columns represent amplification regions and rows represent tumor samples. The yellow color indicates that the region is amplified in the corresponding tumor samples. Columns are sorted by their chromosome locations. Figure 2 shows that the amplification patterns of columns 9, 10, 12 are clustered around that of column 11. Although GISTIC found four separate regions, it seems that the amplifications in columns 9, 10, 12 are not separate events from the amplification in the column 11. The fact that column 11 contains a well known driver gene MYC while the other columns 9, 10, 12 do not contain such genes also support this claim. Therefore, we removed such satellite chromosomal regions and also removed the regions altered in less than or equal to ten samples which leaves 14 driver regions.

Then, we applied our model to the selected driver genes/regions. For each sample *i*, we obtain the posterior mean of the age of tumor lineage *T*_*i*_ and the time of driver gene/region alteration *X*_*i*,*j*_. In Additional file 2: Table S1, we present these values with their 90% confidence interval (CI) obtained by performing 400 bootstraps.

The average value of the posterior mean of tumor ages is 1113 cell divisions. The 10*th* percentile is 563 and the 90*th* percentile is 1839 cell divisions. We removed the gene TP53 from this analysis since it is mutated in almost all samples (95%) and with very few samples in which TP53 is not mutated, it is difficult to estimate the parameters for TP53 correctly. This may have caused the overestimation of the age of tumor lineage since we ignored the possible increase of the alteration rate by the mutation of TP53. Note that the estimated age of tumor lineage is inversely proportional to the alteration rate.

#### Identified mutators

We estimated the increase of mutation rate $\Delta_{j}^{\text{MUT}}$ and CNA rate $\Delta_{j}^{\text{CNA}}$ by the alteration of the gene/region *j* and also obtained their 90% CI from 400 bootstraps. The genes BRCA1, BRCA2 and the chromosomal region 16q23.1 are estimated to increase the mutation rate by 30*%*,50*%* and 120%, respectively. However, only BRCA1 and BRCA2 have 90% CIs which do not include zero. Therefore, we can say only reliably that BRCA1 and BRCA2 genes increase mutation rate. They are well known mutator genes that play key roles in repairing double-strand breaks in DNA [20].

The chromosomal regions 8p21.2, 8q24.21, 16q23.1, 19q12, 22q13.33 are estimated to increase the CNA rate by 70*%*,30*%*,40*%*,30*%* and 50%, respectively. Only the region 8p21.2 and 22q13.33 have 90% CI that do not include zero, implying only they increase CNA rate.

The region 8p21.2 (chromosome 8 between 26165916 bp and 26284094 bp) includes 12 genes, one of which is a tumor suppressor gene PPP2R2A. PPP2R2A is frequently deleted or downregulated in prostate, breast, lung and thyroid cancer [21]. Kalev *et al.*[22] recently revealed that PPP2R2A plays a critical role in double strand break repair through dephosphorylation of ATM. Moreover, they idenfied PPP2R2A as a novel predictive marker for the efficiency of treatment with PARP inhibitors.

The region 22q13.33 (chromosome 22 between 49481137 bp and 49498777 bp) is the most significantly deleted regions of all regions found by GISTIC and all alterations involving this region were telomere loss. The loss of 22q13.33 is the cause of Phelan-McDermid Syndrome characterized by global developmental delay, absent or severely delayed speech, and normal to accelerated growth [23]. Although the role of the deletion of this region in tumorigenesis is not known, telomere loss in general is observed frequently in cancer cells and it is suggested to play an important role in driving the chromosome instability associated with cancer. The telomere loss on the chromosome leads to chromosome fusions between two sister chromatids during mitosis facilitating the accumulation of genetic changes [24,25]. The list of genes included in these regions is provided in the Additional file 1.

Of the four mutator gene/regions selected by our method, three are associated with double strand break repair pathways. The other one is a telomere loss which is known to lead to chromosome instability by chromosome fusion. This provides a degree of validation of our method.

#### Timing of driver alterations

We calculate the posterior mean of the alteration time of each gene/region for each sample *i*. The posterior mean alteration time of the gene/region *j* for the given sample depends on the estimated parameters of the prior distribution for the gene/region, other alterations which occurred in the same sample and the number of passenger alterations in the sample. Table 1 gives the posterior mean alteration time of the gene/region *j* averaged among samples in which *j* is altered and their 90% CIs. Each region is represented by its chromosome location, the candidate target genes included in the region and the type of alteration (amplification or deletion).

**Table 1:** Estimates of the mean time of alteration in cell divisions with its 90% CI from ovarian data

**Gene or region**	**Mean time of alteration**	**90% CI**
	**in cell divisions**	
1p34.2(MYCL1), Amp	307	(67,731)
3q26.2(MECOM), Amp	473	(413,688)
8p21.2(PPP2R2A), Del	6	(0,326)
8q24.21(MYC), Amp	10	(0,383)
10q23.31(PTEN), Del	545	(215,922)
11q14.1(ALG8), Amp	382	(167,830)
12p12.1(KRAS), Amp	62	(47,252)
13q14.2(RB1), Del	256	(196,602)
16q23.1(WWOX), Del	790	(101,851)
17q11.2(NF1), Del	375	(282,637)
19p13.13, Amp	445	(5,729)
19q12(CCNE1), Amp	280	(5,453)
20q13.12(ZMYND8), Amp	111	(81,359)
22q13.33, Del	0	(0,0)
BRCA1	113	(2,132)
BRCA2	2	(0,2)
CSMD3	426	(350,548)
FAT3	338	(288,745)
NF1	177	(73,684)
USH2A	521	(45,690)

The mean time of alteration for each gene/region is calculated by averaging the posterior mean of the alteration time of the gene/region among samples in which it is altered.

**Figure 3:**
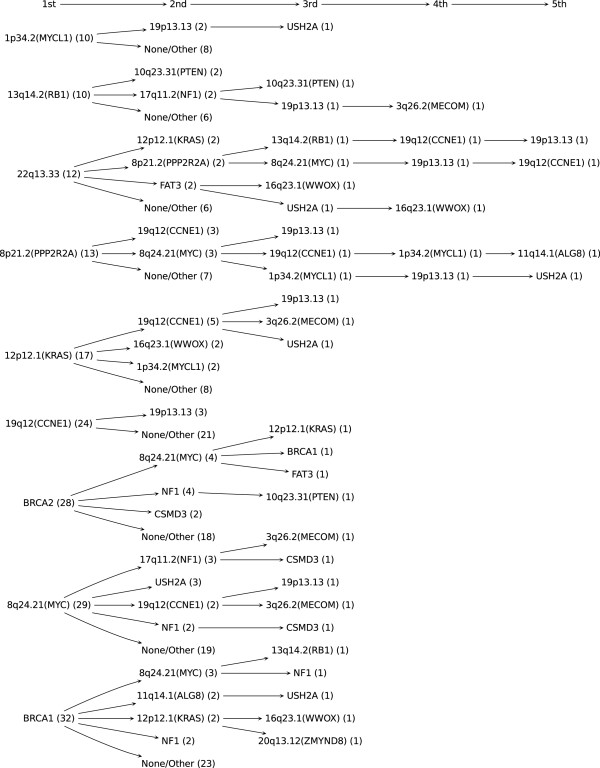
**Order of alterations occurring in ovarian tumor samples represented as a tree structure.** The number in parentheses beside each alteration represents the number of samples which have the same order up to that alteration.

Based on the posterior mean of the alteration time of each gene/region for each sample *i*, we have inferred the order of driver alterations. We estimated the confidence of the sequence by the proportion the same sequence occurred out of 400 sequences obtained from 400 bootstraps. We present the order and its confidence for each sample in Additional file 2: Table S1. Figure 3 shows a summary of the inferred order of alterations occurring in tumor samples represented as a tree structure. The number in parentheses beside each alteration represents the number of samples which have the same inferred order up to that alteration. Figure 3 shows only cases in which the inferred order of the first two driver alterations occurs more than once.

Figure 3 shows that all four mutator gene/regions have the smallest posterior mean time of alterations for most of the samples in which they were altered. One of the main questions in the area of genetic instability in cancer is whether it arises early or late during tumorigenesis. It was suggested that a mutator phenotype would need to be expressed early to generate the causally associated mutations driving tumorigenesis [13], however there has been little evidence supporting this hypothesis. Our result supports the claim that alterations resulting in a mutator phenotype occur early during tumorigenesis. It also shows that in the samples in which the mutator regions 8p21.2 and 22q13.33 are altered, many driver alterations occur afterwards, confirming their roles as mutators.

The non-mutator genes/regions containing MYC, KRAS, CCNE1 and RB1 have the smallest posterior mean time of alterations in most samples while the driver gene CSMD3, USH2A and the region containing MECOM, WWOX have large posterior means.

### Lung cancer data

We applied our method to lung tumor sequencing data from Ding et al. (2008) [26] who sequenced the coding exons and splice sites of 623 candidate cancer genes in 188 samples from patients with lung adenocarcinomas. We applied our method to the driver genes mutated in more than ten samples : APC, ATM, EGFR, KRAS, LRP1B, NF1,PTPRD, STK11, TP53. We also included the gene PRKDC which is mutated in eight samples since it is a well known mutator gene.

In Additional file 3: Table S2, we present the posterior mean of the age of tumor lineage *T*_*i*_ and the alteration time of gene *j*, *X*_*i*,*j*_ with their 90% CI for each sample *i*. The average value of the posterior mean of tumor ages is 749 cell generations. The 10*th* percentile is 236 and the 90*th* percentile is 1617 cell divisions.

#### Identified mutators

We estimated the increase of mutation rate $\Delta_{j}^{\text{MUT}}$ by the alteration of the gene *j* and also obtained their 90% CI from 400 bootstraps. Only two genes, TP53 and PRKDC, were found to increase mutation rates. TP53 increases mutation rate by 170% while PRKDC increases mutation rate by 670%. The 90% CI for $\Delta_{j}^{\text{MUT}}$ of both genes do not include zero.

Both of the genes TP53 and PRKDC are well known mutator genes. A new finding from our method is that PRKDC increases mutation rate much greater than TP53. TP53 activates DNA repair proteins when DNA has sustained damage or it initiates apoptosis if DNA damage is irreparable. PRKDC encodes a protein involved in the repair of double-stranded DNA breaks.

#### Timing of driver alterations

**Figure 4:**
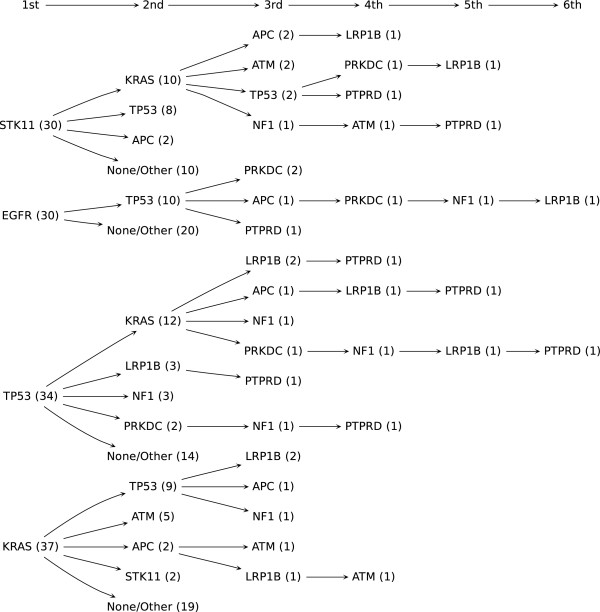
**Order of alterations occurring in lung tumor samples represented as a tree structure.** The number in parentheses beside each alteration represents the number of samples which have the same order up to that alteration.

We have inferred the order of driver alterations by the posterior mean of the alteration time of each gene for each sample *i*. We present the inferred order of driver mutations and its confidence for each sample in Additional file 3: Table S2. The posterior mean alteration time of gene *j* averaged among samples in which *j* is altered and their 90% CIs are given in Table 2. Figure 4 shows a summary of the inferred order of mutations occurring in tumor samples represented as a tree structure.

**Table 2:** Estimates of the mean time of alteration in cell divisions with its 90% CI for the driver genes from lung data

**Gene**	**Mean time of alteration in cell divisions**	**90% CI**
APC	379	(44,801)
ATM	594	(93,805)
EGFR	23	(18,93)
KRAS	280	(158,392)
LRP1B	549	(443,917)
NF1	505	(60,744)
PRKDC	466	(324,1637)
PTPRD	801	(394,1228)
STK11	259	(108,455)
TP53	323	(208,456)

The mean time of alteration for each gene is calculated by averaging the posterior mean of the alteration time of the gene among samples in which it is altered.

It shows that EGFR, TP53, KRAS and STK11 have the smallest posterior mean time of alterations for most of the samples in which they were altered. In our analysis with ovarian cancer data, KRAS amplification was also identified as an early event.

There is much evidence supporting the finding that alterations of KRAS, EGFR, STK11 and TP53 are early events in many cancer types [9]. Figure 4 also shows that LRP1B and PTPRD tend to have the largest posterior mean time of alterations for most of the samples in which they were altered. This suggests that these genes may play important roles in invasion or metastasis. This is supported by the study suggesting LRP1B may be involved in cellular invasion/metastasis [27] and the study showing the association between deletion of PTPRD and cutaneous squamous cell carcinoma metastasis [28].

## Conclusions

We have developed a method which estimates the age of the tumor lineage and the timing of the driver alterations. This method also identifies mutator genes and estimates the increase in rate of alterations caused by the mutator gene during tumorigenesis. We applied this method to TCGA ovarian cancer and lung cancer data. For ovarian cancer data, we used both sequencing and copy number data and found that BRCA1 and BRCA2 increase the rate of point mutations and the chromosomal regions 8p21.2 and 22q13.33 increase the rate of copy number alterations. We found that alterations in genes/regions resulting in a mutator phenotype tend to occur early. For the non-mutator genes/regions, the regions containing MYC, KRAS, CCNE1 and RB1 tend to alter early while the gene CSMD3, USH2A and the region containing MECOM, WWOX tend to alter late.

For lung data, we applied this method to only sequencing data and found that TP53 and PRKDC increase mutation rate. We found that EGFR, KRAS, STK11 and TP53 tend to mutate early while LRP1B and PTPRD tend to mutate late.

This is the first attempt to identify genes that increase the mutation rate or CNA rate using computational methods. Finding mutator genes simply based on the correlation between the number of passenger alterations and the alteration status of a driver gene generates many false positives since it cannot distinguish a mutator gene and a gene that alters late. For both genes, there are high correlations between the number of passenger alterations and their alteration status. For example, if we test for each driver *j* whether there is a difference in the mean between the number of passenger alterations in samples in which driver *j* is altered and those in other samples, we find that LRP1B, NF1, PRKDC, PTPRD, TP53 genes have p values less than 0.01 for lung sequencing data. For ovarian sequencing data, we found that 16q23.1, 19q12, BRCA2, FAT3, USH2A have p values less than 0.01. For ovarian copy number data, we found that 8p21.2, 8q24.21, 16q23.1, 19p13.13, 19q12, 22q13.33 have p values less than 0.01. Note that this method finds many more mutator candidates compared to our method while missing an important mutator BRCA1. The mutator candidates found by the correlation, such as PTPRD or LRP1B whose p-values are 2·10^−6^ and 3·10^−5^ are estimated to be simply altered late by our method. There is no evidence supporting their role in increasing genomic instability, implying they could be false positives.

It is well known that genomic instability can be caused by dysfunction of DNA repair genes and cell cycle checkpoint control genes. The DNA repair genes which have been found to be altered in cancers include BRCA1/2, MSH2/6, MLH1/2, BLM, RAD50, MRE11, NBS1, PRKDC, NBS1, BLM, RECQL4, BAP1, WRN, RAD51L3, RAD52, FANCA, and PALB2 [10-12]. Of the mutator genes identified in our analysis of lung and ovarian cancer, BRCA1/2, PRKDC, and PPP2R2A gene in the region 8p21.2 belong to this category although the role of PPP2R2A in inducing chromosomal instability in ovarian cancer was previously unknown. Other DNA repair genes are rarely altered in our dataset. The genes in the cell cycle checkpoint control pathway which have been found to be altered in cancers include TP53, ATM, MDM2/4, BUB1, and STK12. Of the mutator genes we identified, TP53 belongs to this category.

In addition to the DNA repair and cell cycle chekpoint processes, there are many other processes involved in genomic stability. These include DNA replication, deoxynucleotide metabolism, chromosome condensation, sister chromatid cohesion, kinetochore structure and function and centrosome/microtubule formation. Therefore, in principle, there are many genes that could induce genomic instability. Other than these processes, telomere erosion is known to be able to lead to chromosome instability. In our analysis of ovarian data, we found that the deletion of 22q13.33 is telemere loss which leads to chromosome instability. This is a new finding that supports the role of telomere erosion in CIN of ovarian cancer.

Our method also provides an estimate of tumor age and timing of driver alterations which can be obtained only through computational methods. The age of the tumor lineage is the number of cell divisions in the dominant clone’s lineage from the birth of the patient until the biopsy. Some tissues such as pancreatic epithelia do not self-renew, therefore, most of the cell divisions in the lineage of the pancreatic tumor occur after the onset of neoplasia [29]. Therefore the age of the tumor lineage corresponds approximately to the tumor age, the time interval from the onset of neoplasis to the tumor detection in units of cell generations. Some tissues such as skin or gastrointestinal epithelia regulary self-renew. In these cases, the number of cell divisions in the lineage is the sum of the number of cell divisions before the onset of neoplasia and that after the onset of neoplasia. If the cell division rate has been constant throughout a life, the age of the tumor lineage corresponds to the age of the patient. In this paper, we estimated the average age of the tumor lineage for the ovarian tumor is 1113 cell divisions and that for the lung tumor is 749 cell generations. Ovarian elithelia regularly self renew [29], while lung epithelia renew slowly and are stimulated to self-renew upon injury [30], therefore, the age of the tumor lineage for lung tumor is close to the tumor age. The cell division time for a lung tumor cell is known to be approximately 8 days [31]. Therefore it takes 749·8 days =16.4 years on average from the beginning of the tumor to the detection of lung tumor.

Estimates of tumor age, together with clinical data such as tumor stage can provide information for how long it takes for a benign tumor to develop into invasive and metastatic tumors. Estimating when metastasis occurs during tumorigenesis is particularly important since metastasis is responsible for most cancer related deaths although it is the least understood process. Understanding this can help planning early detection programs for cancer since it is critical to know how early you have to detect the tumor in order to have an effect. If the tumor metastasizes before detection, then early detection of the primary tumor may not help the patient. For example, cancer screening has been successful for both colon and cervical cancers in reducing death rate but results for breast cancer are less successful, indicating that screening breast mammography fails to detect cancer until after they have spread [32]. Although this problem of estimating when metastasis occurs has not been dealt with in this paper, it is an important future work that our method can be used to answer.

Estimates of tumor age also provide insight into the biology of tumor cell populations, may help to understand intra-tumor heterogeneity and differences in prognosis and responsiveness to therapy. A previous attempt to estimate the tumor age using the number of passenger mutations [2] did not take into account the increase of the mutation rate by the alteration of mutator genes, and hence their estimate of tumor age may be somewhat overestimated. We believe our method will be a useful contribution for better understanding the process of tumorigenesis.

## Availability of supporting data

The software and data are available at: https://sites.google.com/site/ahrimy2013/home/software.zip.

## Competing interests

The authors declare that they have no competing interests.

## Authors’ contributions

AY and RS conceived the study. AY implemented the algorithm. AY and RS wrote the manuscript. Both authors read and approved the final manuscript.

## Supplementary Material

Additional file 1 Supplementary Materials. Click here for file

Additional file 2 Table S1. Click here for file

Additional file 3 Table S2. Click here for file
