# Supplementary material for: Using passenger mutations to estimate the timing of driver mutations and identify mutator alterations
Source: BMC Bioinformatics. 2013 Dec 13;14:363. doi: 10.1186/1471-2105-14-363 (PMC3903072; doi:10.1186/1471-2105-14-363)
Supplement: Additional file 1 — Supplementary Materials. [file 1471-2105-14-363-S1.pdf]

Supplementary Materials

for

Using Passenger Mutations to Estimate the Timing of Driver Mutations and Identify Mutator Alterations

Ahrim Youn Richard Simon

Probability Model

The founder cell of the major clone traces a genealogy back to the fertilized egg. From the fertilized egg, passenger somatic alterations are accumulated in the lineage of the founder cell. The accumulation of passenger somatic alterations can be modeled by a Poisson process with rate λ per cell division if the alteration rate stays constant. (Figure 1 (a)) In order to permit variation among patients, we model the number of passenger somatic alterations N_i as following a Poisson distribution with rate $\lambda T_i + E_i$ where T_i is the number of cell divisions in the lineage of the founder cell from the birth to the tumor detection for sample i and E_i is the increase of alterations by unknown factors such as exposure to mutagens by smoking or UV radiation.

We assume an alteration of a driver gene or driver region j increases the alteration rate by Δ_j. It is positive if the driver is a mutator and 0 otherwise. Suppose the alteration of the driver gene or region j occurred in sample i at time $X_{i,j}$. Then the alteration rate per cell division is λ until the time $X_{i,j}$ and after that, it becomes $\lambda + \Delta_j$. Therefore, the number of passenger alterations follows a Poisson distribution with rate $\lambda X_{i,j} + (\lambda + \Delta_j)(T_i - X_{i,j}) + E_i = \lambda T_i + \Delta_j(T_i - X_{i,j}) + E_i$. (Figure 1 (b))

Now, suppose another driver gene or region k is altered in sample i at time $X_{i,k}$. If $X_{i,j} < X_{i,k}$, the alteration rate is λ by $X_{i,j}$, $\lambda + \Delta_j$ between $X_{i,j}$ and $X_{i,k}$ and $\lambda + \Delta_j + \Delta_k$ afterwards. Therefore the number of passenger alterations follows a Poisson distribution with rate $\lambda X_{i,j} + (\lambda + \Delta_j)(X_{i,k} - X_{i,j}) + (\lambda + \Delta_j + \Delta_k)(T_i - X_{i,k}) + E_i = \lambda T_i + \Delta_j(T_i - X_{i,j}) + \Delta_k(T_i - X_{i,k}) + E_i$. The same result is derived when $X_{i,k} < X_{i,j}$. This means that the alteration of each driver gene or region j increases the average number of passenger alterations accumulated in the sample by $\Delta_j(T_i - X_{i,j})$ additively. (Figure 1 (c))

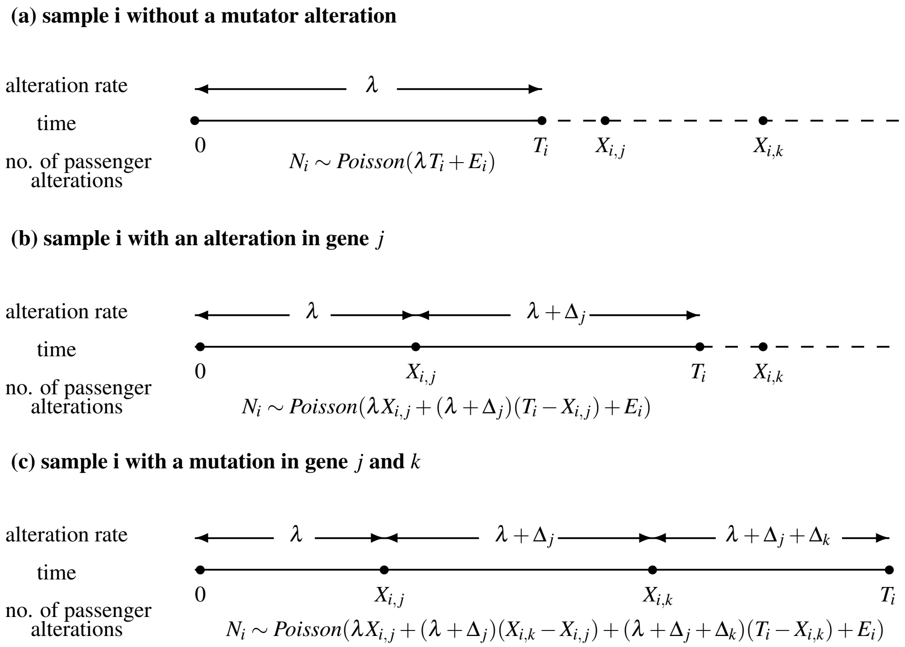

λ = basic alteration rate per cell division

N_i = number of passenger alterations in sample i

T_i = number of cell divisions in the lineage of the tumor founder cell from the birth to the biopsy

$X_{i,j}$ = time at the alteration of gene j in units of cell divisions

Δ_j = increase of the alteration rate per cell division caused by an alteration of gene j

E_i = increase of total alteration rate by unknown factors

Figure 1: Probability Model for generation of passenger somatic alterations

Derivation of the likelihood

The likelihood element of observing the number of passenger somatic alterations N_i and the occurrences of alterations $A_{i,j}$ of selected driver genes or regions $j \in J$ given their germline or somatic status $G_{i,j}$ and age of the patient S_i is,

$$
\begin{aligned}
& \Pr(N_i = n_i, A_{i,j} = a_{i,j}, \forall j \in J \,|\, G_{i,j} = g_{i,j}, \forall j \in J, S_i = s_i) \\
= \;& \int_{t_i=50}^{s_i/r} \underset{x_{i,j}=(0,\infty)\ \forall j\in J_i}{\int\cdots\int} \Pr(N_i = n_i, T_i = t_i, A_{i,j} = a_{i,j}, X_{i,j} = x_{i,j}, \forall j \in J_i \,|\, G_{i,j} = g_{i,j}, \forall j \in J, S_i = s_i) \prod_{j\in J_i} \mathrm{d}x_{i,j}\, \mathrm{d}t_i \quad (J_i = J \cap \{j \,|\, g_{i,j} = 0\}) \\
= \;& \int_{t_i=50}^{s_i/r} \underset{x_{i,j}=(0,\infty)\ \forall j\in J_i}{\int\cdots\int} \Pr(N_i = n_i, A_{i,j} = a_{i,j}, X_{i,j} = x_{i,j} \forall j \in J_i \,|\, T_i = t_i, G_{i,j} = g_{i,j}, \forall j \in J) f(T_i = t_i | S_i = s_i) \prod_{j\in J_i} \mathrm{d}x_{i,j}\, \mathrm{d}t_i \\
= \;& \int_{t_i=50}^{s_i/r} \underset{x_{i,j}=(0,\infty)\ \forall j\in J_i}{\int\cdots\int} \Pr(N_i = n_i, A_{i,j} = a_{i,j}, \forall j \in J_i \,|\, T_i = t_i, X_{i,j} = x_{i,j}, \forall j \in J_i, X_{i,h} = 0, \forall h \in J - J_i) \prod_{j\in J_i} f(X_{i,j} = x_{i,j})\, \mathrm{d}x_{i,j}\, f(T_i = t_i | S_i = s_i)\, \mathrm{d}t_i \\
= \;& \int_{t_i=50}^{s_i/r} \underset{x_{i,j}=(0,\infty)\ \forall j\in J_i}{\int\cdots\int} \Pr(N_i = n_i, x_{i,k} \leqq t_i, \forall k \in K_i, x_{i,l} > t_i, \forall l \in L_i \,|\, T_i = t_i, X_{i,j} = x_{i,j}, \forall j \in J_i, X_{i,h} = 0, \forall h \in J - J_i) \prod_{j\in J_i} f_{X_{i,j}}(x_{i,j})\, \mathrm{d}x_{i,j}\, f_{T_i}(t_i | S_i = s_i)\, \mathrm{d}t_i \\
& (K_i = J_i \cap \{j \,|\, a_{i,j} = 1\}, L_i = J_i \cap \{j \,|\, a_{i,j} = 0\}) \\
= \;& \int_{t_i=50}^{s_i/r} \underset{\substack{x_{i,k}=(0,t_i)\ \forall k\in K_i \\ x_{i,l}=[t_i,\infty]\ \forall l\in L_i}}{\int\cdots\int} \int_{e_i=0}^{\infty} \frac{(\sum_{k\in K_i} \Delta_k(t_i - x_{i,k}) + (\lambda + \sum_{h\in J-J_i} \Delta_h)t_i + e_i)^{n_i} e^{-\sum_{k\in K_i} \Delta_k(t_i - x_{i,k}) - (\lambda + \sum_{h\in J-J_i} \Delta_h)t_i - e_i}}{n_i!} f_{E_i}(e_i)\, \mathrm{d}e_i \prod_{j\in K_i \cup L_i} f_{X_{i,j}}(x_{i,j})\, \mathrm{d}x_{i,j}\, f_{T_i}(t_i | S_i = s_i)\, \mathrm{d}t_i \\
= \;& \int_{t_i=50}^{s_i/r} \underset{x_{i,k}=(0,t_i)\ \forall k\in K_i}{\int\cdots\int} \int_{e_i=0}^{\infty} \frac{(\sum_{k\in K_i} \Delta_k(t_i - x_{i,k}) + (\lambda + \sum_{h\in J-J_i} \Delta_h)t_i + e_i)^{n_i} e^{-\sum_{k\in K_i} \Delta_k(t_i - x_{i,k}) - (\lambda + \sum_{h\in J-J_i} \Delta_h)t_i - e_i}}{n_i!} f_{E_i}(e_i)\, \mathrm{d}e_i \prod_{k\in K_i} f_{X_{i,k}}(x_{i,k})\, \mathrm{d}x_{i,k} \prod_{l\in L_i} (1 - F_{X_{i,l}}(t_i))\, f_{T_i}(t_i | S_i = s_i)\, \mathrm{d}t_i \\
= \;& \int_{t_i=50}^{s_i/r} \underset{x_{i,k}=(0,t_i)\ \forall k\in K_i'}{\int\cdots\int} \int_{e_i=0}^{\infty} \frac{(\sum_{k\in K_i'} \Delta_k(t_i - x_{i,k}) + (\lambda + \sum_{h\in J-J_i} \Delta_h)t_i + e_i)^{n_i} e^{-\sum_{k\in K_i'} \Delta_k(t_i - x_{i,k}) - (\lambda + \sum_{h\in J-J_i} \Delta_h)t_i - e_i}}{n_i!} f_{E_i}(e_i)\, \mathrm{d}e_i \prod_{k\in K_i'} f_{X_{i,k}}(x_{i,k})\, \mathrm{d}x_{i,k} \prod_{m\in K_i - K_i'} F_{X_{i,m}}(t_i) \prod_{l\in L_i} (1 - F_{X_{i,l}}(t_i))\, f_{T_i}(t_i | S_i = s_i)\, \mathrm{d}t_i \\
& (K_i' = K_i \cap \{j \,|\, \Delta_j > 0\})
\end{aligned}
$$

where $f_{X_{i,j}}(x_{i,j}) = p_j \frac{\beta_j^{\alpha_j}}{\Gamma(\alpha_j)} x_{i,j}^{\alpha_j - 1} e^{-\beta_j x_{i,j}}$, $f_{E_i}(e_i) = \rho e^{-\rho e_i}$, $f_{T_i}(t_i | S_i = s_i) = \frac{1}{G_{\alpha,\beta}(s_i/r) - G_{\alpha,\beta}(50)} \frac{\beta^\alpha}{\Gamma(\alpha)} t_i^{\alpha-1} e^{-\beta t_i}$ ($G_{\alpha,\beta}$ is a cumulative Gamma distribution with shape and rate parameters α, β).

The difficult part of the calculation is the multiple integration over $X_{i,k}$ for $k \in K_i'$. However it can be converted into a sum of products of single integrals as follows :

$$
\begin{aligned}
& \underset{x_{i,k}=(0,t_i)\ \forall k\in K_i'}{\int\cdots\int} \int_{e_i} \frac{(\sum_{k\in K_i'} \Delta_k(t_i - x_{i,k}) + (\lambda + \sum_{h\in J-J_i} \Delta_h)t_i + e_i)^{n_i} e^{-\sum_{k\in K_i'} \Delta_k(t_i - x_{i,k}) - (\lambda + \sum_{h\in J-J_i} \Delta_h)t_i - e_i}}{n_i!} f_{E_i}(e_i)\, \mathrm{d}e_i \prod_{k\in K_i'} f_{X_{i,k}}(x_{i,k})\, \mathrm{d}x_{i,k} \\
= \;& \underset{x_{i,k}=(0,t_i)\ \forall k\in K_i'}{\int\cdots\int} \int_{e_i} \frac{1}{n_i!} \sum_{m + \sum_{k\in K_i'} m_k \le n_i} \frac{n_i!}{(n_i - m - \sum_{k\in K_i'} m_k)!\, m!\, \prod_{k\in K_i'} m_k!} e_i^m \prod_{k\in K_i'} (\Delta_k(t_i - x_{i,k}))^{m_k} ((\lambda + \sum_{h\in J-J_i} \Delta_h)t_i)^{n_i - m - \sum_{k\in K_i'} m_k} e^{-\sum_{k\in K_i'} \Delta_k(t_i - x_{i,k}) - (\lambda + \sum_{h\in J-J_i} \Delta_h)t_i - e_i} f_{E_i}(e_i)\, \mathrm{d}e_i \prod_{k\in K_i'} f_{X_{i,k}}(x_{i,k})\, \mathrm{d}x_{i,k} \\
= \;& \underset{x_{i,k}=(0,t_i)\ \forall k\in K_i'}{\int\cdots\int} \int_{e_i} \frac{1}{n_i!} \sum_{m + \sum_{k\in K_i'} m_k \le n_i} \frac{n_i!}{(n_i - m - \sum_{k\in K_i'} m_k)!\, m!\, \prod_{k\in K_i'} m_k!} \prod_{k\in K_i'} ((\Delta_k(t_i - x_{i,k}))^{m_k} e^{-\Delta_k(t_i - x_{i,k})} f_{X_{i,k}}(x_{i,k})\, \mathrm{d}x_{i,k}) ((\lambda + \sum_{h\in J-J_i} \Delta_h)t_i)^{n_i - m - \sum_{k\in K_i'} m_k} e^{-(\lambda + \sum_{h\in J-J_i} \Delta_h)t_i} e_i^m e^{-e_i} f_{E_i}(e_i)\, \mathrm{d}e_i \\
= \;& \underset{x_{i,k}=(0,t_i)\ \forall k\in K_i'}{\int\cdots\int} \int_{e_i} \sum_{m + \sum_{k\in K_i'} m_k \le n_i} \prod_{k\in K_i'} (\frac{1}{m_k!} (\Delta_k(t_i - x_{i,k}))^{m_k} e^{-\Delta_k(t_i - x_{i,k})} f_{X_{i,k}}(x_{i,k})\, \mathrm{d}x_{i,k}) \frac{1}{(n_i - m - \sum_{k\in K_i'} m_k)!} ((\lambda + \sum_{h\in J-J_i} \Delta_h)t_i)^{n_i - m - \sum_{k\in K_i'} m_k} e^{-(\lambda + \sum_{h\in J-J_i} \Delta_h)t_i} \frac{1}{m!} e_i^m e^{-e_i} f_{E_i}(e_i)\, \mathrm{d}e_i \\
= \;& \sum_{m + \sum_{k\in K_i'} m_k \le n_i} \prod_{k\in K_i'} \int_{x_{i,k}=0}^{t_i} (\frac{1}{m_k!} (\Delta_k(t_i - x_{i,k}))^{m_k} e^{-\Delta_k(t_i - x_{i,k})} f_{X_{i,k}}(x_{i,k})\, \mathrm{d}x_{i,k}) \frac{1}{(n_i - m - \sum_{k\in K_i'} m_k)!} ((\lambda + \sum_{h\in J-J_i} \Delta_h)t_i)^{n_i - m - \sum_{k\in K_i'} m_k} e^{-(\lambda + \sum_{h\in J-J_i} \Delta_h)t_i} \int_{e_i} \frac{1}{m!} e_i^m e^{-e_i} f_{E_i}(e_i)\, \mathrm{d}e_i
\end{aligned}
$$

$$
\begin{aligned}
&= \sum_{m+\sum\limits_{k\in K_i'} m_k \le n_i} \prod_{k\in K_i'} \int_{x_{i,k}=0}^{t_i} \left(\frac{1}{m_k!}(\Delta_k(t_i-x_{i,k}))^{m_k} e^{-\Delta_k(t_i-x_{i,k})} f_{X_{i,k}}(x_{i,k})\,\mathrm{d}x_{i,k}\right) \frac{1}{(n_i-m-\sum\limits_{k\in K_i'} m_k)!} ((\lambda+\sum_{h\in J-J_i}\Delta_h)t_i)^{n_i-m-\sum\limits_{k\in K_i'} m_k} e^{-(\lambda+\sum\limits_{h\in J-J_i}\Delta_h)t_i} \int_{e_i} \frac{\rho}{m!} e_i^m e^{-(1+\rho)e_i}\,\mathrm{d}e_i \\
&= \sum_{m+\sum\limits_{k\in K_i'} m_k \le n_i} \prod_{k\in K_i'} \int_{x_{i,k}=0}^{t_i} \left(\frac{1}{m_k!}(\Delta_k(t_i-x_{i,k}))^{m_k} e^{-\Delta_k(t_i-x_{i,k})} f_{X_{i,k}}(x_{i,k})\,\mathrm{d}x_{i,k}\right) \frac{1}{(n_i-m-\sum\limits_{k\in K_i'} m_k)!} ((\lambda+\sum_{h\in J-J_i}\Delta_h)t_i)^{n_i-m-\sum\limits_{k\in K_i'} m_k} e^{-(\lambda+\sum\limits_{h\in J-J_i}\Delta_h)t_i} \int_{e_i} \frac{\rho}{m!} \frac{\Gamma(m+1)}{(\rho+1)^{m+1}} \frac{(\rho+1)^{m+1}}{\Gamma(m+1)} e_i^m e^{-(1+\rho)e_i}\,\mathrm{d}e_i \\
&= \sum_{m+\sum\limits_{k\in K_i'} m_k \le n_i} \prod_{k\in K_i'} \int_{x_{i,k}=0}^{t_i} \left(\frac{1}{m_k!}(\Delta_k(t_i-x_{i,k}))^{m_k} e^{-\Delta_k(t_i-x_{i,k})} f_{X_{i,k}}(x_{i,k})\,\mathrm{d}x_{i,k}\right) \frac{1}{(n_i-m-\sum\limits_{k\in K_i'} m_k)!} ((\lambda+\sum_{h\in J-J_i}\Delta_h)t_i)^{n_i-m-\sum\limits_{k\in K_i'} m_k} e^{-(\lambda+\sum\limits_{h\in J-J_i}\Delta_h)t_i} \frac{\rho}{(\rho+1)^{m+1}} \\
&= \sum_{\sum\limits_{k\in K_i'} m_k \le n_i} \prod_{k\in K_i'} \int_{x_{i,k}=0}^{t_i} \left(\frac{1}{m_k!}(\Delta_k(t_i-x_{i,k}))^{m_k} e^{-\Delta_k(t_i-x_{i,k})} f_{X_{i,k}}(x_{i,k})\,\mathrm{d}x_{i,k}\right) \frac{1}{(n_i-\sum\limits_{k\in K_i'} m_k)!} ((\lambda+\sum_{h\in J-J_i}\Delta_h)t_i)^{n_i-\sum\limits_{k\in K_i'} m_k} e^{-(\lambda+\sum\limits_{h\in J-J_i}\Delta_h)t_i} \sum_{m\le n_i-\sum\limits_{k\in K_i'} m_k} \frac{(n_i-\sum\limits_{k\in K_i'} m_k)!((\lambda+\sum\limits_{h\in J-J_i}\Delta_h)t_i)^{-m}}{(n_i-m-\sum\limits_{k\in K_i'} m_k)!} \frac{\rho}{(\rho+1)^{m+1}}
\end{aligned}
$$

Parameter estimation

The unknown values of the parameters $\alpha, \beta, \alpha_j, \beta_j, \Delta_j, p_j, \rho$ are estimated by maximizing the likelihood

$$\prod_i \Pr(N_i, A_{i,j}, \forall j \in J \mid G_{i,j}, \forall j \in J, S_i).$$

Before calculating the maximum likelihood estimates (MLE), we first test whether ρ is infinite or not. This is equivalent to testing whether $E_i = 0$ for all samples i or not. Note that E_i follows an exponential distribution with the parameter ρ, and therefore when ρ is infinite, E_i is always zero. We do a likelihood ratio test of ρ being infinite vs. not. When the null hypothesis is not rejected under the p-value cutoff 0.05, we fix the value of ρ as infinite and estimate other parameters by maximizing the likelihood. If the null hypothesis is rejected, we estimate the value of ρ and other parameters by maximizing the likelihood.

When estimating the parameters α_j, β_j for a gene/region j, we found that there is not a unique MLE for some genes/regions. In those cases, for the given value of β_j, there is an interval of α_j which gives the same likelihood. In such cases, we find the interval of α_j giving the same maximum likelihood and give the median value of the interval as the estimate of α_j.

Preselection of mutator genes

The calculation and optimization of the likelihood becomes difficult for a large set of drivers J. Therefore, we predetermine the set of mutators whose Δ_j value is positive and only include these mutators in J and estimate $\alpha, \beta, \alpha_j, \beta_j, \Delta_j, p_j, \rho$ by maximizing the likelihood. This is because non-mutators do not increase the rate of alterations and thus do not affect the estimates α, β, ρ as well as $\alpha_j, \beta_j, \Delta_j, p_j$ of other genes.

For non-mutators k, $\Delta_k = 0$. The parameters α_k, β_k, p_k of non-mutators are estimated by maximizing

$$\prod_i \Pr(A_{i,k} \mid G_{i,k}, N_i, S_i, A_{i,j}, G_{i,j}, \forall j \in J)$$

using the estimates obtained above.

To predetermine the set of non-mutators whose Δ_k value is zero, we fit a model which includes only one driver gene/region k. Then we obtain an estimate of Δ_k and its 95% confidence interval (CI) by doing 100 bootstraps. If the 95% CI for Δ_k includes 0, we consider the gene as non-mutator.

For bootstrap calculations, we utilized the high-performance computational capabilities of the Biowulf Linux cluster at the National Institutes of Health, Bethesda, Md. (http://biowulf.nih.gov).

Bootstrapping

For the sensitivity analysis of parameter estimates, we did 400 bootstrappings. For each bootstrap, we randomly sampled tumor samples (of equal size to the orginal dataset) with replacement from the original dataset. We applied our method to obtain MLEs of parameters for each bootstrapped dataset. Therefore we obtain 400 sets of parameter estimates. Using these, we construct confidence intervals for Δ_j, the posterior mean of T_i and $X_{i,j}$. When calculating MLEs for each bootstrapped data, we used the MLEs obtained from the original data as the starting value for the optimization of the likelihood. This may have biased the confidence interval to be narrow.

List of genes belonging to mutator CNA regions

Table 3: List of genes belonging to the CNA region 8p21.2 and 22q13.33

CNA region	Genes
8p21.2 (Chr 8 : 26165916-26284094)	PPP2R2A,KCTD9,BNIP3L,GNRH1,CDCA2,DPYSL2, DOCK5, PNMA2,EBF2,NEFL,ADRA1A,NEFM
22q13.33 (Chr 22 : 49481137-49498777)	SHANK3,RABL2B,ACR,RPL23AP82

Comparison with the previous method of mutation order estimation

We compared the result for the lung data with the result for the same data obtained by our previous method of the order estimation [1]. It estimates $P_{k,i}$, the probability that the $k'th$ mutational event involving the selected driver genes occurs in gene i. Table 4 shows the estimates of $P_{k,i}$ and their 90% confidence interval. For better comparison of the results from both methods, we also present the conditional probability that the gene mutates early (a gene mutates at the $k'th$ event for $k \leq 3$) or late (a gene mutates at the $k'th$ event for $k > 3$) given that the gene is mutated in the sample and their 90% CIs in Table 5. The conditional probabilities clarify whether a gene mutates early or late. Table 6 shows the result obtained by our current method. Although it is difficult to compare the results because each method uses different measures for ordering, both identify EGFR, KRAS, STK11, TP53 as early mutating genes and LRP1B, NF1, PRKDC, PTPRD as late mutating genes.

References

[1] Youn, A., Simon, R.: Estimating the order of mutations during tumorigenesis from tumor genome sequencing data. Bioinformatics **28**(12), 1555–1561 (2012)

Table 4: Estimates of $P_{k,i}$ for lung tumors

Gene	$P_{1,i}$		$P_{2,i}$		$P_{3,i}$		$P_{4,i}$		$P_{5,i}$	
	MLE	90% CI	MLE	90% CI	MLE	90% CI	MLE	90% CI	MLE	90% CI
APC	0	(0 , 0.02)	0.05	(0.01 , 0.14)	0.21	(0 , 0.48)	0	(0 , 0.5)	0.16	(0 , 0.67)
ATM	0.01	(0 , 0.02)	0.14	(0.09 , 0.25)	0	(0 , 0.2)	0	(0 , 0.36)	0.13	(0 , 0.5)
EGFR	0.22	(0.16 , 0.26)	0	(0 , 0)	0	(0 , 0.03)	0	(0 , 0)	0	(0 , 0)
KRAS	0.43	(0.37 , 0.51)	0	(0 , 0)	0	(0 , 0.58)	0	(0 , 0)	0	(0 , 0)
LRP1B	0.03	(0.01 , 0.09)	0	(0 , 0)	0.2	(0 , 0.6)	0.57	(0 , 1)	0	(0 , 0.66)
NF1	0.06	(0.03 , 0.11)	0	(0 , 0.02)	0.09	(0 , 0.29)	0	(0 , 0.38)	0.35	(0 , 0.67)
PRKDC	0.02	(0.01 , 0.05)	0	(0 , 0)	0.11	(0 , 0.35)	0	(0 , 0.34)	0.35	(0 , 0.75)
PTPRD	0	(0 , 0.04)	0.05	(0.01 , 0.13)	0.1	(0 , 0.49)	0.43	(0 , 0.88)	0	(0 , 0.79)
STK11	0.14	(0.08 , 0.19)	0.09	(0.03 , 0.21)	0.29	(0.13 , 0.6)	0	(0 , 0.37)	0	(0 , 0.92)
TP53	0.09	(0.05 , 0.14)	0.67	(0.57 , 0.76)	0	(0 , 0)	0	(0 , 0)	0	(0 , 0)

Table 5: Probabilities that observed mutations occur early* or late* for lung tumors

Gene	Early	90% CI	Late	90% CI
APC	0.44	(0.09 , 1)	0.74	(0 , 0.98)
ATM	0.33	(0.1 , 1)	0.78	(0 , 0.98)
EGFR	1	(1 , 1)	0	(0 , 0)
KRAS	1	(1 , 1)	0	(0 , 0)
LRP1B	0.34	(0.08 , 1)	0.85	(0 , 1)
NF1	0.18	(0.06 , 1)	0.95	(0 , 0.99)
PRKDC	0.16	(0.02 , 1)	0.96	(0 , 1)
PTPRD	0.29	(0.12 , 1)	0.83	(0 , 0.96)
STK11	1	(1 , 1)	0	(0 , 0.46)
TP53	1	(1 , 1)	0	(0 , 0)

*early means $1_{st}, 2_{nd}$ or 3_{rd} event and late means later events

Table 6: Estimates of the mean time of alteration in cell divisions with its 90% CI for the driver genes from lung data.

Gene	mean time of alteration in cell divisions	90% CI
APC	379	(44 , 801)
ATM	594	(93 , 805)
EGFR	23	(18 , 93)
KRAS	280	(158 , 392)
LRP1B	549	(443 , 917)
NF1	505	(60 , 744)
PRKDC	466	(324 , 1637)
PTPRD	801	(394 , 1228)
STK11	259	(108 , 455)
TP53	323	(208 , 456)
